# 

**Published:** 1885-12

**Authors:** 


Please Send Your Subscription
for 1886.

Vol. VI. DECEMBER, 1885. No. 12.

LIBRARY
INDEXED
S.G.O

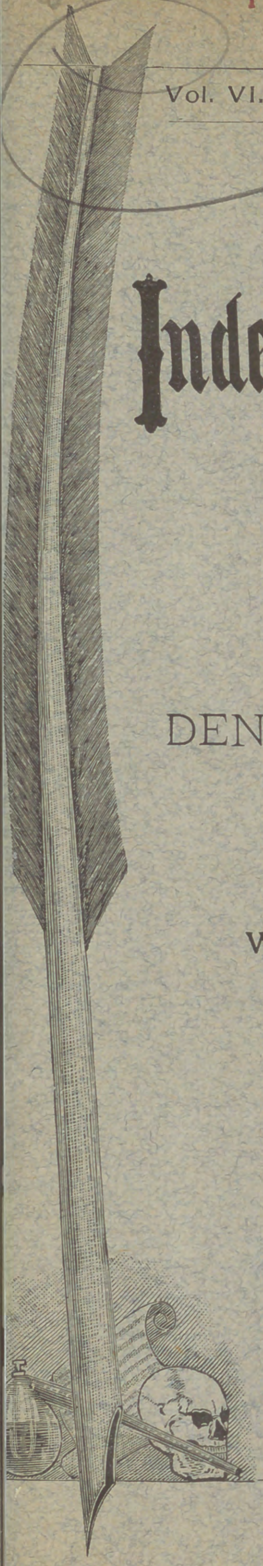

### THE
### Independent Practitioner:

### A MONTHLY JOURNAL

DEVOTED TO

### DENTAL AND ORAL SCIENCE.

---

W. C. BARRETT, M. D., D. D. S.,
EDITOR.

---

THE NEW YORK DENTAL JOURNAL ASSOCIATION,
PUBLISHERS,
No. 35 West 46th Street, New York.
AND
No. 208 Franklin St., Buffalo, N. Y.

$2.50 Per Annum in Advance, - - - - Single Copies, 25 Cents.

Entered at the Post-Office, Buffalo, N. Y., as Second-Class matter.

